# Characterizing a clinically significant radiographic parastomal hernia recurrence: post hoc analysis of a randomized controlled trial

**DOI:** 10.1007/s00464-025-11988-8

**Published:** 2025-08-11

**Authors:** William C. Bennett, Sara M. Maskal, Andrew S. Conner, Kimberly P. Woo, Pavel Lenkov, Alvaro C. Carvalho, Daphne Remulla, Ryan C. Ellis, Kimberly S. Miles, Chao Tu, Benjamin T. Miller, Lucas R. Beffa, David M. Krpata, Ajita S. Prabhu, Michael J. Rosen, Clayton C. Petro

**Affiliations:** 1 Cleveland Clinic Foundation, Cleveland, OH USA; 2 Cleveland Clinic Lerner College of Medicine, Case Western Reserve University School of Medicine, Cleveland, OH USA; 3 Northwestern Medical Center, Chicago, IL USA

**Keywords:** Parastomal, Hernia, Recurrence

## Abstract

**Introduction:**

Parastomal hernia recurrence is common but poorly defined. We aimed to correlate radiographic recurrence and reoperation for parastomal hernia recurrence with patient-reported outcomes (PROs) to determine the clinical significance of radiographic recurrence and identify potential screening tools.

**Methods:**

Patient data in the one- and two-year follow-up windows of a registry-embedded randomized controlled trial comparing open retromuscular Sugarbaker and keyhole mesh parastomal hernia repairs (2019–2022) were analyzed. CT scan images were classified using the Moreno-Matias (MM) classification scheme and PROs were analyzed for trends across groups. PROs included stoma-specific quality of life (Colostomy Impact Score – CIS, range 0–38), pain (NIH PROMIS 3a, range 30.7–71.8), abdominal wall-specific quality of life (HerQLes, range 0–100), and the Decision Regret Scale (DRS, range 0–100) in reference to the previous repair.

**Results:**

Of 150 randomized patients, there were 221 cross-sectional images and 5 reoperations for recurrence with time-matched PROs. Radiographic recurrence classification ordinally correlated with worse stoma-specific quality of life (CIS, *p* = 0.0001), with a significant difference first noted between MM 0 and MM II (*p* = 0.02). Reoperation for recurrence was associated with both worse stoma-specific quality of life (CIS, *p* = 0.02) and pain (PROMIS 3a, *p* = 0.02). Abdominal wall-specific quality of life and decision-regret were not associated with radiographic recurrence or reoperation.

**Conclusion:**

Radiographic MM classification appears to be clinically meaningful with regard to stoma-specific quality of life. Significant differences were first observed at MM II, supporting the use of MMII as a cutoff to define a clinically significant parastomal hernia recurrence. Additionally, measurements of stoma-specific quality of life and pain are potential screening mechanisms to identify patients that should have cross-sectional imaging and counseling on reoperation.

**Graphical Abstract:**

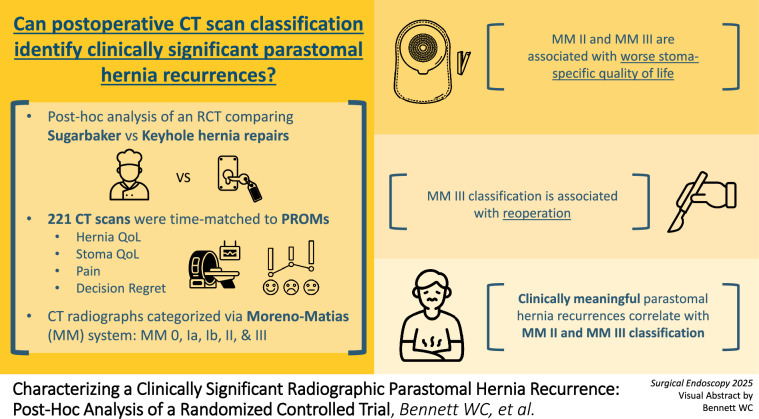

**Supplementary Information:**

The online version contains supplementary material available at 10.1007/s00464-025-11988-8.

Parastomal hernia (PSH) is a common complication of permanent stoma creation, sometimes considered an inevitability given rates as high as 50%[1–3], though a true rate remains elusive given variability in follow-up and PSH definition. PSH repair is cautiously offered given subsequent recurrence rates as high as 76% [4]. Guidance regarding diagnosis and management of recurrent PSH (rPSH) is scant. Patient quality of life (QoL) outcomes of rPSH, while well-understood regarding primary PSH, have been poorly described. The Moreno-Matias (MM) and European Hernia Society (EHS) classifications are the most utilized PSH classification systems in contemporary literature, however, their significance in identifying a clinically significant rPSH has not been reported. Further, there exists almost no literature regarding thresholds to operate on rPSHs, but the largest series of parastomal hernia repairs to-date suggests 20% of patients will undergo reoperation for recurrence after an index PSH repair [5].

This post hoc analysis of an RCT comparing recurrence rates at 2 years between retromuscular (RM) Sugarbaker vs RM keyhole repairs for PSH aimed to contribute to literature characterizing rPSH as a clinical entity. Follow-up imaging captured within 2 post-operative years was categorized by MM classification. Trends in reoperation and time-matched patient-reported outcomes (PRO) measures were considered across MM types to investigate the clinical significance of MM classification and radiographic recurrence after RM PSH repair.

## Methods

This is a post hoc analysis of a randomized clinical trial comparing the efficacy of the RM Sugarbaker vs keyhole mesh configuration in reducing parastomal hernia recurrence at 2 years. The protocol was registered on ClinicalTrials.gov (NCT03972553) and a post hoc analysis was approved by our institutional review board. Details of the trial have been previously reported [6]. Briefly, patients aged 18 years or greater with a permanent stoma and associated parastomal hernia requiring open repair were enrolled and randomized to RM Sugarbaker or keyhole PSH repair from April 2019 to April 2022. Patients with > 2 stomas, insufficient bowel length for either repair technique, or non-mesh reconstructions were excluded. The primary outcome was pragmatic hernia recurrence at 2 years as determined by blinded consensus review of radiographic imaging, clinical examination, or patient-reported bulge. Patients underwent cross-sectional imaging (CT or MRI) without Valsalva maneuver, clinical exam, and PRO administration at 1 year (± 4 months) and 2 years (± 6 months). Radiographic recurrence was originally defined in a binary fashion consistent with MM classifications II and III (hernia sac containing non-stoma bowel viscera) [7]. For the purposes of this study, images were further classified by the MM classification scheme (0, Ia, Ib, II, III). Patient-reported outcomes (PROs) included the short-form NIH Patient-Reported Outcomes Measure Information System Pain Intensity Scale (PROMIS 3a v1.0) [8], abdominal wall-specific quality of life using the Hernia-Related Quality-of-Life Survey (HerQLes) [9], stoma-specific quality of life using the Colostomy Impact score (CIS) [10], and decision regret regarding the choice to undergo parastomal hernia repair using the Decision Regret Scale (DRS) [11] at annual follow-up intervals.

Data from the RCT were used to compare PROs against MM classification groups to identify trends in patient QOL. MM classification and PROs were also considered across patients who underwent reoperation vs those who did not to characterize differences perhaps informing reoperation decision making.

### Patient identification, data collection, and image review

Trial data were reviewed to identify sets of imaging studies and time-matched PROs within the 1- or 2-year follow-up windows. Patient charts were reviewed to ensure images remained accessible. If new eligible radiographic series were identified (CT or MRI images within a follow-up window with PROs), the image temporally closest to the PRO was utilized after verifying no relevant interventions occurred between the image and PRO. Locations of imaging series within the electronic medical record (EMR) were sent to at least three of six qualified surgeons who participated in the original trial (A.S.P., D.M.K, L.R.A.B., B.T.M, M.J.R., C.C.P.) to identify the MM classification of each; classification was determined in a majority-rules consensus and images failing to reach consensus were discussed until unanimous agreement. If the first two responses from participant surgeons agreed, the third surgeon’s response was not sought or captured. For the purposes of inter-rater reliability testing this absent data was treated as missing completely at random and not imputed. Additionally, for scans requiring discussion and unanimous agreement, the original raters’ classifications remained the same for the kappa analysis and the remaining three surgeons’ ratings classifications matched the group consensus. This was similarly chosen to avoid agreement inflation which could occur if all surgeons’ classifications were changed to the consensus decision.

The MM system categorizes degree of parastomal herniation (or lack thereof) across five designations: MM 0 (no herniation), MM Ia (hernia sac with stoma loop, less then 5 cm at greatest width), MM Ib (hernia sac with stoma loop, greater than or equal to 5 cm at greatest width), M II (sac containing omentum), and MM III (sac containing bowel loop other than stoma) [7]. Surgeons were blinded to randomization and prior determination of recurrence.

Patient demographics, operative characteristics, stoma characteristics, clinical post-operative outcomes, and PROs were obtained from two registries utilized in the trial— a study-specific REDCap database and the Abdominal Core Health Quality Collaborative (ACHQC).

### Patient-reported outcome surveys

The PROMIS 3a instrument is a 3-question numeric survey regarding pain severity over the preceding seven days, reported as T scores of 30.7 to 71.8, wherein higher scores indicate greater pain [8]. The HerQLes comprises 12 Likert-scale questions regarding abdominal wall quality of life scored from 0 to 100, with higher scores indicating better quality of life, and a minimum clinically important difference of 15.6 points [9, 12]. The DRS is scored from 0 to 100, with higher scores indicating greater regret [11]. The CIS is a seven item questionnaire validated in patients with permanent colostomies following rectal cancer resection and scored from 0 to 38 points; higher scores indicating worse stoma-related quality of life and scores greater than or equal to 10 are indicative of a “major colostomy impact” [10]. The item regarding fecal consistency was excluded for patients with urostomy, with a subsequent score range of 0 to 30.

Not all patients received or completed every PRO at each interval. CIS was added after the onset of the trial and is missing from some records. Some patients were lost to follow-up; further, follow-up (imaging and PROs) was not routinely captured for patients who underwent operative manipulation of the stoma (even if unrelated to hernia recurrence) or patients who met the primary endpoint (recurrence) prior to the 2-year window.

### Statistical analysis

All complete image-PRO sets were pooled (agnostic to post-operative year) prior to the initial analysis, as the outcomes of interest considered trends between PROs and degree of recurrence on imaging which are unrelated to time from surgery. For the analyses comparing reoperative and non-reoperative patients, patients from the original pool were divided accordingly. Descriptive statistics were generated to produce mean, standard deviation, median, and interquartile range (IQR). Comparisons of continuous variables between two groups utilized unpaired Welch’s t tests when data was normally distributed or Mann–Whitney test otherwise; continuous variable comparisons between three or more groups were conducted utilizing nonparametric Kruskal–Wallis analysis of variance testing followed by Dunn’s multiple comparisons tests with GraphPad Prism version 10.4.1 for macOs (GraphPad Software; Boston, MA; www.graphpad.com). Categorical comparisons across two or more groups were conducted via Chi-Square test of independence. Fisher’s exact test was used in lieu of Chi-Square test for groups with fewer than 5 subjects. Statistical significance was predetermined at p-value < 0.05. Inter-rater reliability was calculated utilizing a two-sided Krippendorff’s alpha test as this accommodates a variable number of raters per item, ordinal distance weights, and missing ratings; a quadratic weight matrix was specified to penalize disagreements between distant MM grades more heavily than adjacent disagreements. Cohen’s *d* effect sizes were computed by subtracting comparator groups’ mean score from the MM Ib group’s and dividing the difference by the pooled standard deviation of the two groups (pooled SD calculated via Bessel’s correction, *df* = *n*₁ + *n*₂ − 2). Inter-rater reliability and Cohen’s *d* effect size analyses were conducted in R (version 4.4.0) utilizing *irr*, *irrCAC, tidyverse*, and *effsize* libraries.

## Results

Of 150 participants randomized in the trial, 130 (86.7%) patients had at least one image-PRO set, and 96 of these (73.8%) had image-PRO sets at both annual intervals for total 221 included sets. One hundred thirteen (51%) sets were from the one-year follow-up window and 108 (49%) sets were from the two-year follow-up window. Of 10 patients who underwent reoperation, five had complete PRO-image sets. Across PRO-image sets, the median age was 66 years (IQR 56, 75), median BMI was 30.1 (27.6, 35.1), 116 (52.5%) identified as female, 114 were randomized to Sugarbaker repair (52%), and the majority of patients had ileostomies (52%), followed by colostomies (30%), then urostomies (18%). Median time between imaging and index operation was 16 months (11, 21). Demographic and operative data are summarized in Table 1. A majority of images were classified as MM 0 (*n* = 135, 61%), 23 (10.4%) were classified as MM Ia, 23 (10.4%) as MM Ib, 13 (5.8%) as MM II, and 27 (12.2%) as MM III; baseline characteristics between groups were similar (Supplement 1).

**Table 1:** Patient demographics and baseline clinical characteristics

	n or Median	% or IQR
Age	66	56, 75
BMI	30.1	27.6, 35.1
Stoma type		
Ileostomy	115	52.04%
Colostomy	67	30.32%
Urostomy	39	17.65%
Gender		
Female	116	52.49%
Male	105	47.51%
Race		
Black or African American	5	2.26%
Hispanic	2	0.9%
White	214	96.83%
ASA Class		
2	10	4.52%
3	201	90.95%
4	10	4.52%
Taking immunosuppressants		
No	196	88.69%
Yes	25	11.31%
Current nicotine use		
No	201	91.78%
Yes	18	8.22%
HTN		
No	71	32.13%
Yes	150	67.87%
DM		
No	180	81.45%
Yes	41	18.55%
COPD		
No	195	88.24%
Yes	26	11.76%
Post-Op year		
1 year (± 6 m)	113	51.13%
2 year (± 6 m)	108	48.87%
Time from operation (months)	16	11, 21
Prior randomization		
Sugarbaker	114	51.58%
Keyhole	107	48.42%

### Moreno-Matias classification and PROs

**Fig. 1:**
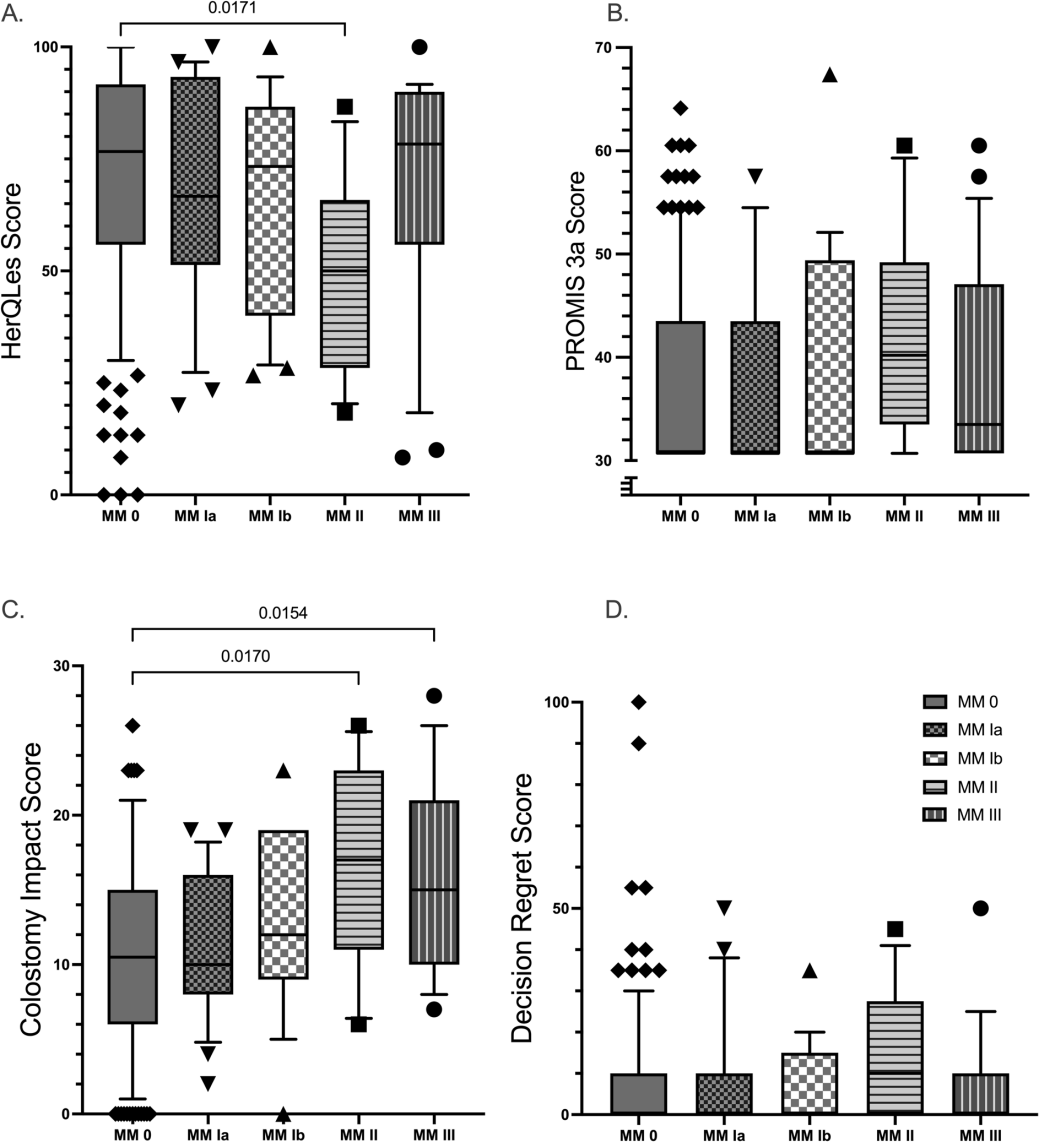
Box-and-whiskers representation of survey scores across Moreno-Matias groups. Whiskers represent 10th and 90th percentiles. Pairwise statistically significant differences are demonstrated (a = 0.05)

Median HerQLes score was 73.33 (IQR 50.33, 91.67) across groups (Fig. 1A). A statistical difference was appreciated across groups (*p* = 0.0272); however, a linear trend was not observed, and post hoc testing identified only a difference between MM II (median 50; IQR 28.3, 65.8) and MM 0 (median 76.67; IQR 55.83, 91.67, *p* = 0.0253) correlating with increased quality of life for patients with no detectable recurrence on radiography (Fig. 1A). Differences in pain via PROMIS 3a score (median 30.7, IQR 30.7, 46.30) were not observed across groups (Fig. 1B). The lowest possible score of 30.7 was the median for MM 0, MM Ia, and MM Ib; with MM II (median 40.20) and MM III (median 33.50) demonstrating no meaningful elevation between classification groups (*p* = 0.4074).

Stoma-related quality of life as measured by CIS was, however, different across MM classification groups (*p* = 0.0010). Median score across all groups was 11.50 (IQR 7, 17), which is notably above the threshold score of 10 for “major life impact,” and scores generally increased across groups (Fig. 1C). Post hoc comparisons demonstrated statistically significant group-to-group differences between MM 0 (median 10.5; IQR 6, 15) and both MM II (median 17; IQR 11, 23; *p* = 0.025) and MM III (median 15; IQR 10, 21; *p* = 0.0288).

Decision regret had no detectable differences across groups (*p* = 0.2195) (Fig. 1D). All groups had patients with “high regret” scores of greater than or equal to 25 (14% of MM 0, 17% of MM Ia, 9% of MM Ib, 30% of MM II, and 11% of MM III, but the proportion of these across groups was also not significant (*p* = 0.449).

Further, in a subgroup analysis comparing scores across years, there were no detectable differences in HerQLes (*p* = 0.0905), PROMIS 3a (*p* = 0.246), CIS (*p* = 0.2725), or DRS (*p* = 0.7866) to suggest improvement or deterioration of QoL indices across the 1- or 2-year time points.

### Reoperative group

Three of the five reoperative patients with PROs had images categorized as MM III, one was MM II and one was MM 0, with no reoperations occurring in patients with MM Ia or MM Ib classifications. This corresponded with an 11.1% reoperation rate for MM III-classified recurrences (Table 2A). Of note, the MM 0 patient underwent reoperation for frequent prolapse, but the patient’s imaging (without Valsalva) was unanimously classified as MM 0 both at 1-year and 2-year intervals.

**Table 2:** Reoperation rates for recurrence/revision stratified by Moreno-Matias classification

MM Class	Patients requiring reoperation (%)		
A. original inclusion/exclusion criteria			
MM 0 (*n* = 135)	1	(0.74%)	p = 0.0071
MM Ia (*n* = 23)	0	(0.00%)	
MM Ib (*n* = 23)	0	(0.00%)	
MM II (*n* = 13)	1	(7.69%)	
MM III (*n* = 27)	3	(11.11%)	
B. Including reoperations without PROs			
MM 0 (*n* = 135)	1	(0.74%)	p < 0.0001
MM Ia (*n* = 23)	0	(0.00%)	
MM Ib (*n* = 24)	1	(4.17%)	
MM II (*n* = 13)	1	(7.69%)	
MM III (*n* = 31)	7	(22.58%)	

When considering all 10 reoperations (including five patients with images but no corresponding PROs), seven (70%) recurrences requiring reoperation were MM III, one was MM II, one was MM Ib, none were MM Ia, and one was MM 0 (Table 2B; *p* < 0.0001).

**Fig. 2:**
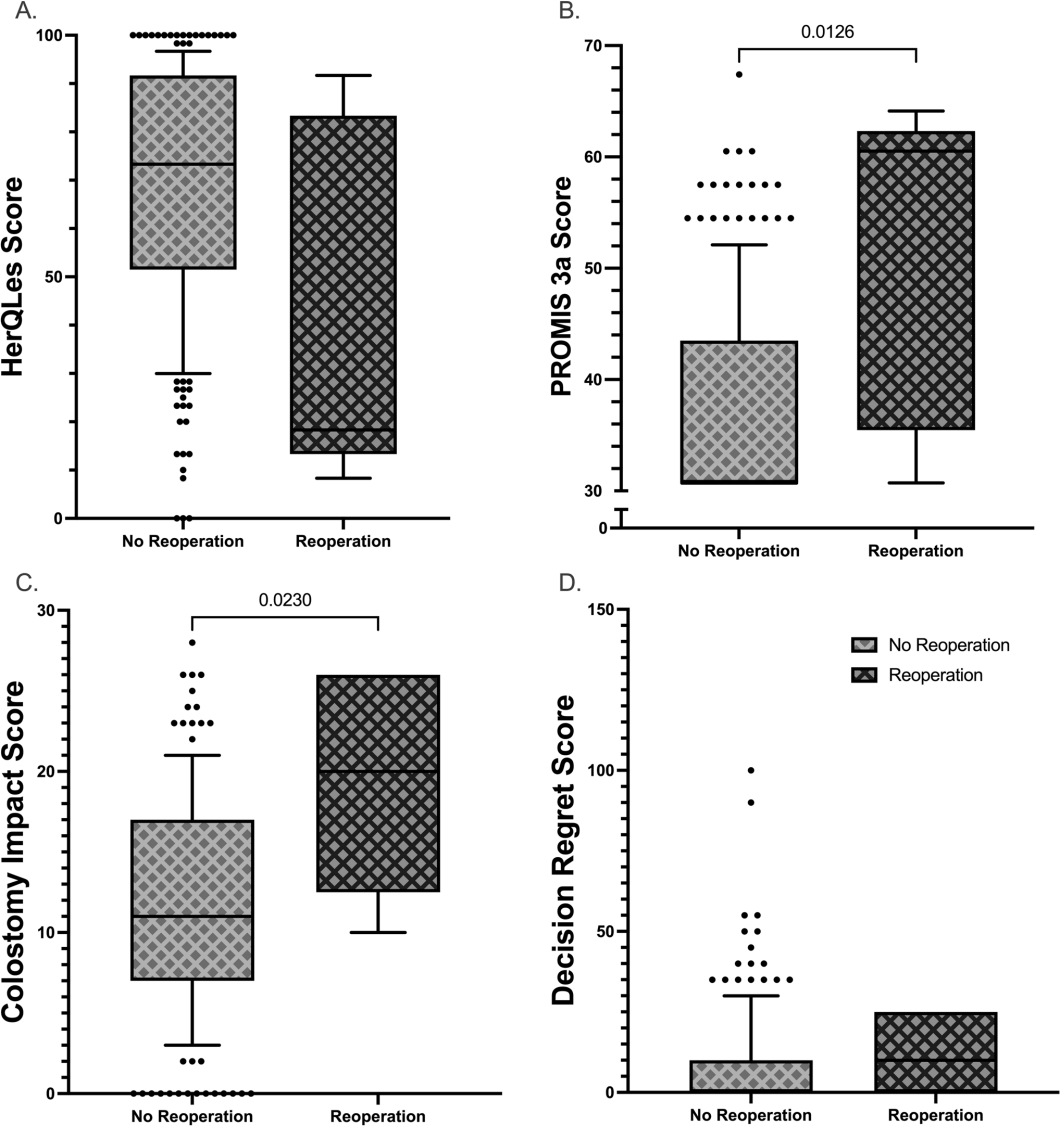
Box-and-whiskers representation of survey scores across reoperation groups. Whiskers represent 10th and 90th percentiles. Pairwise statistically significant differences are demonstrated (a = 0.05)

When comparing the five patients with complete image-PRO sets who underwent reoperation vs the 216 who did not, HerQLes score did not differ statistically (median 18.33 vs 73.33, *p* = 0.0938) (Fig. 2A). The PROMIS 3a survey demonstrated a significant difference in pain between groups (*p* = 0.0142) wherein the non-reoperative group had a median of the lowest possible score (30.70; IQR 30.70, 43.50) vs the reoperative groups median of 60.50 (IQR 34.45, 62.20), out of a max score of 67.4 (Fig. 2B). The colostomy impact score was significantly worse in those requiring reoperation (*p* = 0.0230); with a reoperation group median of 20 (IQR 12.5, 26), nearly twice that of the non-reoperative group median (11; IQR 7, 17). Once again, both groups had a median score greater than the threshold for “high impact.” Similar to consideration of MM classification, differences in decision regret score were no different between reoperation and non-reoperation groups (median 10 vs 0, p = 0.304).

### Evaluation of MM Ib or MM II as recurrence cutoff

When defining recurrence by the original MM definition (including MM Ib as recurrent) vs the MM Ib-exclusive definition utilized in our trial, PROs support the exclusive definition regarding patient outcomes. While multi-group comparison of HerQLes scores demonstrated a significant difference across groups, subsequent pairwise comparisons demonstrated this was largely driven by the abrupt decrease in quality of life of MM II and its difference from the MM 0 group. In contrast, MM Ib exhibited the second highest quality of life. Negative impact of stoma on QoL via the CIS, however, was clearly associated with increasing size. Both MM II and MM III demonstrated significant differences from MM 0, but a difference was not observed between MM 0 and MM Ia nor MM Ib. Given this finding, ‘recurrent’ and ‘non-recurrent’ groups were formed with assignment of MM Ib to the non-recurrent group and difference in CIS scores were investigated. This analysis demonstrated a persistent difference between the CIS scores of the recurrent and non-recurrent groups (median 16 vs 11, *p* = 0.00015), agreeing with the MM Ib-exclusive recurrence definition (Table 3).

**Table 3:** Analysis of Moreno-Matias Ib-exclusive definition of recurrence

Outcome measure	MM 0 – MM Ib			MM II – MM III			p
	n	Median	IQR	n	Median	IQR	
HRQLS (range: 0–100)	175	75	51.67, 91.67	39	66.67	51.67, 86.67	0.055
PROMIS 3a (range: 30.7–71.8)	176	30.7	30.7, 43.5	39	40	30.7, 46.3	0.124
CIS (range: 0–38)	180	11	6, 16	40	16	10.25, 21	0.0001^§^
DRS (range: 0–100)	181	0	0, 10	40	5	0, 18.75	0.074
Reoperations for recurrence/revision, n (%)	2 of 182 (1.23%)			8 of 44 (18.2%)			< 0.0001^§§^

^§^Denotes significance via Mann–Whitney test, a = 0.05

^§§^Denotes significance via Fisher’s exact test, a = 0.05

**Fig. 3:**
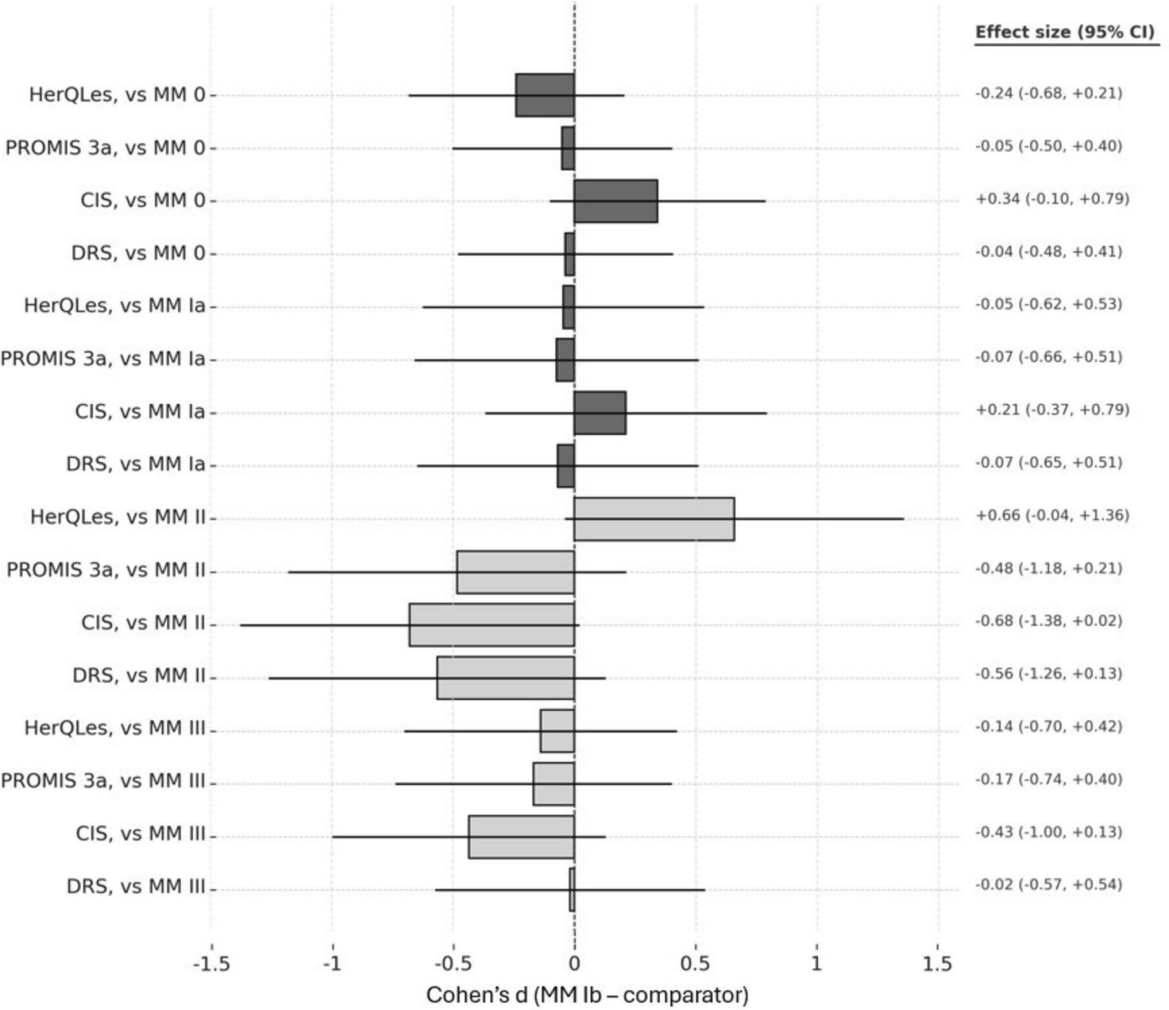
Forest plot of Cohen’s d between MM Ib and other MM groups across each patient-reported outcome measure. Positive values indicate higher scores in MM Ib relative to the comparator and negative scores indicate lower MM Ib scores. Magnitudes of 0.2, 0.5, and 0.8 SD were predetermined to represent small, medium, and large differences, respectively

Cohen’s effect size difference testing also suggested greater similarity between the non-recurrent groups (MM 0 and MM Ia) and MM Ib vs the recurrent groups (MM II and MM III; Fig. 3). Though no MM classifications were statistically different from MM Ib due to limited sample sizes, as demonstrated previously, the effect size differences of the recurrent groups were greater than the non-recurrent groups.

### Inter-rater agreement

A total of 685 surgeon classifications were available for 221 CT scans. The marginal distribution of grades was MM0–51%, MM Ia – 16%, M Ib – 15%, MM II–7%, and MM III–11%. Overall reliability was high with Krippendorff’s α of 0.99 (95% CI 0.997–1.000) and the nominal (unweighted) α was similar (0.998). To assess potential inflation of inter-rater agreement due to the “stop-when-two-agree” procedure, we conducted a second analysis on the 162 scans which received exactly three initial ratings, for which the α remained 0.998.

## Discussion

Our data demonstrate correlations with enlarging MM classification and stoma-specific QoL. Specifically, image series rated as MM II were associated with a statistically significant difference in hernia-specific quality of life and stoma-specific quality of life vs non-recurrent (MM0) series. Reoperations for parastomal hernia recurrences were found to be related to stoma-specific quality of life and pain among patients who completed 1-year and/or 2-year follow-up after open retromuscular PSH repair. Together, these results align with primary PSH literature regarding size/classification, symptoms, and decisions around (re)operative intervention. While other studies almost exclusively consider primary PSH, this data provides key insights into the interpretation of radiographic studies, reoperative decision-making, and patient experiences regarding recurrent PSH following open RM repair.

### MM classification and quality of life

The association between MM classification and a readily available QoL instrument is an important finding for multiple reasons. CT scans are frequently utilized in rPSH surveillance. Routine radiographic follow-up is often standard of care in clinical practice and research protocols; thus, clinical correlations with radiographic findings are essential to justify utilization of the technology and appropriately interpret findings [1, 6]. However, beyond characterization of a patient’s outcome as ‘herniation/recurrence’ or ‘no herniation/non-recurrent’ when summarizing post-operative PROs across groups, scant literature correlates symptom severity across a continuum of PSH sizes/radiographic classifications. Though the original MM system was designed to correlate imaging and exam findings with patient-reported symptoms, the determination of symptomatic vs asymptomatic was binary [7]. While patients with a PSH classified as MM II might have experienced greater symptom severity vs MM Ib, the system was not designed to account for such differences. No studies to-date have evaluated differences in symptom severity across each MM classification; recently, the largest systematic review of clinic-radiological accuracy of PSH was unable to identify literature associating radiographic recurrence classification with symptom severity [13]. While one study has validated the preoperative European Hernia Society Classification System of parastomal hernias (EHSC) for operative planning and captured post-operative QoLs, they did not report preoperative PROs, preventing stratification of symptom profiles across EHSC types [14].

Thus, the observation that MM correlates with the widely available CIS instrument is an important step in supporting the clinical relevance of the MM system. Our findings suggest utility for CIS as a potential post-operative surveillance screening tool for patients who could benefit from imaging to assess recurrence, regardless of stoma type. Further the observation of greater life impact for patients with MM II and MM III as determined by CIS, can inform surgeons’ interpretation of radiographs for patients with rPSH.

However, the observation of a higher median CIS score in the MM II vs MM III group, while not statistically significant, merits attention. Limitations in the utilization of a non-validated questionnaire or inadequate sample size could explain this; but the finding could also be inherent to the MM classification’s disregard of defect size and the static nature of CT scans. Patients with scans demonstrating MM II (herniation of non-bowel contents into the sac, namely, omentum), may actually have a defect aperture which accommodates bowel herniation while standing, prone, or with Valsalva (though not seen on imaging). As such, some literature has advocated for Valsalva or prone positioning to be part of all PSH radiographic protocols [15]; notably, though, the MM classification was validated only in supine patients without Valsalva [7]. For such patients, the risk of intermittent bowel obstruction may be the same or higher than MM III. Similarly, the presumably smaller defect of MM II PSH (vs MM III) may incur greater congestion at the defect neck as additional omentum herniates through, leading to partial obstruction of the stoma or omental infarction, both of which could induce discomfort or stoma dysfunction.

### Quality of life and reoperation

Patient-reported pain and stoma burden on life were significantly elevated in patients receiving reoperation after RM PSH repair vs those without reoperation. Quality of life is a central consideration when offering patients parastomal hernia repair, and no guidelines currently provide recommendations for watchful waiting vs surgical management (regarding primary or recurrent PSH) [4]. However, the threshold to electively operate on rPSH is anecdotally higher than primary hernias. Recurrent PSH repair is associated with a higher rate of wound morbidity and recurrence [16, 17], which has been shown to increase with each subsequent repair [18]. While our study did not capture PROs following reoperation (as patients were said to have met the primary endpoint of the index trial upon receiving a reoperation for recurrence), this would be an important step for future efforts.

Our data does, however, make direct comparisons regarding patient-reported quality of life between patients with recurrence at the time when reoperative decision-making occurred, identifying factors which likely prompted consideration of reoperation. When considering the magnitude of difference between reoperative and non-reoperative groups, the median non-reoperative PROMIS3a T score of 30.70 is the lowest possible on the scale, whereas the median reoperative score of 60.50 is the 4th highest (for PROMIS3a v1.0). Pain, as it relates to PSH, has been discussed previously and multiple studies have identified a correlation between patient-reported generalized pain and onset of PSH [19–21]. By implementing multiple QoL surveys featuring pain, Krogsgaard et al. [22], appeared to isolate stoma site pain as the primary driver of this association. Only one study has previously demonstrated a link between recurrence and pain, wherein 7 of 77 patients recurred after mesh-based PSH repair and all 3 patients with new onset chronic pain were in the recurrence group [23]. In our study, PROMIS 3a score did not differ across MM groups, nor in our analysis of the MM Ib-exclusive recurrence definition. Instead, our results suggest a role for PROMIS3a in post-operative surveillance of PSH— not to detect recurrence, but to identify patients who may require reoperation.

As with MM classification group, our results associated CIS with reoperation. Notably, however, the median score in both patient groups (non-reoperative and reoperative) was greater than 10 (11 vs 22, respectively) indicating a “major” life impact of the stoma. Given our other results demonstrating the utility of CIS in capturing negative outcomes of PSH, it is not surprising that the CIS score was higher in patients who ultimately required reoperation. However, that the median score for all patients was above the threshold for “major life impact” was unexpected. Timing of data collection may have contributed to this finding. The CIS was initially validated with survey results from patients at a mean 4.6 years following stoma creation [10]. Patient ‘response shifts’ over time have been demonstrated to influence QoL scores, with trends toward improvement over time [24, 25]. In contrast to this, one study failed to identify a difference in QoL scores at < 12 months vs > 12 months after stomal surgery; however, their mean follow-up for the > 12 month group was not reported [26]. We did not observe a meaningful difference between patients in the 1 year vs 2 year groups regarding any of the QoL measures, and any further analysis of a time-dependent effect is outside the scope of this investigation. Alternatively, the increased discomfort/burden could be attributed to the RM repair with PCS. While the technique [6] and early surgical outcomes [27] have been previously reported in literature, our study represents the only study to-date reporting patient QoL following RM SB and KH repairs and this finding may be novel. As such, further investigation is required to characterize patient-reported outcomes in addition to surgical outcomes, which have been given far more attention [28–32].

### Recurrence and reoperation — can imaging guide management?

For the purpose of defining PSH as a research outcome— whether primary PSH after the utilization of prophylactic mesh or rPSH following repair— current literature is variable. In a 2020 systematic review [13], only 62% of studies included a definition, and among these 19 different definitions were identified. Eight of these were based on radiographic findings alone. Classification was only considered in 45% of studies, most of which utilized MM, then the EHSC, and a minority featured both. The MM classification system both defines and classifies PSH using imaging modalities that are now widely available in clinical practice, making it a useful instrument in research.

However, the current MM definition of a “true hernia,” and thus recurrent hernias following repair, may diagnose PSH recurrence in patients without clinically significant symptoms. The rate of PSH recurrence is known to be high, but a minority of patients undergo reoperation, suggesting excessive sensitivity for recurrence by contemporary diagnostic schema and inadequate specificity for clinically meaningful disease. As mentioned, the MM Ib-inclusive definition of a clinically significant PSH in the original study by Moreno-Matias only regarded a binary determination of ‘symptomatic’ and did not consider symptom severity. However, our data demonstrate worsening stoma-specific quality of life for patients with MM II, rather than MM Ib, and support a stricter definition of recurrence starting at MM II. While no statistically significant differences between MM Ib or other groups were detected (likely due to sample size constraints), MM II was demonstrated to be different from MM 0 regarding abdominal wall-specific QoL and stoma-specific QoL. Further, the subsequent Cohen’s effect size difference analysis suggested less difference between MM Ib and the classically non-recurrent groups (MM 0 and MM Ia) regarding stoma-specific quality of life, pain, and decision regret. Given the limited sample sizes of the non-MM 0 groups, the analysis was underpowered, and the results are not statistically. However, they are highly suggestive of a symptomatic delineation occurring between MM Ib and MM II.

Regarding reoperation— arguably the most salient indicator of a clinically significant recurrence— only recurrences classified as MM II and MM III from the primary dataset were observed to have undergone reoperation for true recurrence. The MM 0-classified reoperation included above was for a prolapsing stoma, which does not meet the majority of accepted definitions of PSH; however, we did not prespecify this exclusion and chose to include it in the final analysis. Notably, there was one patient from the MM 1b cohort who underwent reoperation but was excluded from the initial analysis due to an absence of time-matched PROs. When the reoperative patients without PROs were included, the reoperation rate for MM Ib was 4.17%; far lower than the 22.58% reoperation rate observed for MM III-classified recurrences.

Altogether, our data suggest the clinical significance of a recurrent PSH corresponds with an MM II classification or greater, defined as the point at which cross-sectional imaging detects extraperitoneal displacement of intraperitoneal contents. Patient-reported outcome measures demonstrate a decline in QoL once characterized as MM II, and reoperation rates are significantly elevated for image series with MM III classifications. Thus, an MM II recurrence definition is effective for characterizing and detecting clinically significant PSHs which may benefit from stoma nursing or counseling on possible reoperation.

The utility of MM classification in guiding reoperative decision-making is a novel finding which has not been previously demonstrated [4]. Only a single retrospective study [33], which excluded rPSH, has investigated radiographic PSH classification systems’ with eventual need for operative repair of PSH. They found an association with the EHSC type, but not MM type among 62 patients with PSH, 27% of which underwent reoperation. They observed EHS Type III (no concomitant midline and defect size > 5 cm) PSHs were less likely to undergo reoperation than Type I (no concomitant midline and defect size ≤ 5 cm) PSHs. This finding is loosely discordant with our findings. While MM does not consider defect size, presuming MM III defects are larger than MM II defects, we observed reoperation rate to increase with increasing MM class. This discrepancy could represent differences in group practice patterns as both were single-center studies, decision-making regarding primary PSH vs rPSH, or decision-making following open RM repairs in particular. Further studies stratifying reoperative decision making and outcomes across classifications are certainly required to better understand the predictive utility of these systems.

## Limitations

While our findings provide important data to characterize the clinical presentation of rPSH, numerous limitations should be considered in the interpretation of our analyses. Our analysis was confined to the data collected during the trial. As such, the PROs utilized in the trial— selected in part due to their availability and investigators’ familiarity with these instruments— are not validated for all stoma types, PSH, or rPSH. Our selected stoma-specific quality of life survey (CIS) was initially validated for end colostomies following oncologic resection and while it is now validated for benign disease processes [34–37], it has seldom been utilized for other intestinal stomas in literature. However, our results suggest CIS score detects worsening QoL in patients with increasing rPSH type on radiographic imaging regardless of stoma type, which was internally supported given the statistically significant relationship between CIS and reoperation (a group which featured no colostomies). Replication of these results with stoma-specific QoL instruments validated for specific stoma types will be an important next step in further expanding contemporary literature. Similarly, that we did not segregate any results by stoma type is a weakness. Literature suggests a higher QoL impact for colostomies and ileostomies vs urostomies[26] which could confound interpretation of QoL when we are utilizing this measure to detect the clinical impact of herniation (rather than stoma type).

Regarding logistical constraints, the initial trial design imposed boundaries on this post hoc analysis. Data collection was halted for patients once they met the primary endpoint (recurrence or reoperation for recurrence) or if their stoma was operatively manipulated (for any reason). This not only limited the number of patients with complete imaging and PRO sets but also prevented follow-up of patients who underwent reoperation. Most notably, this culled the reoperative group by half to only 5 subjects with image and matching PROs. This small sample size imposes limits in the power and generalizability of our data, and additional follow-up of these patients should be considered to better characterize the number and features of patients who eventually require reoperation. Given the frequency with which prospective studies generate data that is later analyzed in a post hoc fashion, this is an important consideration when designing future trials. Disagreement between reviewers when determining the MM classification of images is another potential limitation. Kappa analysis demonstrated high inter-rater agreement suggesting internal consistency which provides some mitigation of this concern. Finally, almost all cases in the trial were performed during elective admissions which skews the population away from trends observed in general practice where 6–20% of PSH repairs are performed on a non-elective basis which is associated with increased morbidity and mortality [38].

## Conclusion

The MM classification system corresponds with stoma-related quality of life for patients with rPSH following RM repair. Pain and stoma-related quality of life are associated with reoperation for rPSH, as is MM III classification. An ideal definition of recurrence utilizing MM classifications remains elusive; however, our previously implemented definition of MM II and MM III appears useful for research purposes given the association with reoperation and stoma-related quality of life. Overall, recurrent PSH remains poorly characterized and additional studies considering patient quality of life are merited, especially at longer follow-up windows with disease-specific instruments.

## Supplementary Information

Below is the link to the electronic supplementary material.

Supplementary file1 (DOCX 28 KB)
