# Supplementary material for: Characterizing a clinically significant radiographic parastomal hernia recurrence: post hoc analysis of a randomized controlled trial
Source: Surg Endosc. 2025 Aug 11;39(10):6773–84. doi: 10.1007/s00464-025-11988-8 (PMC12500758; doi:10.1007/s00464-025-11988-8)
Supplement: Supplementary file 1 — Supplementary file1 (DOCX 28 KB) [file 464_2025_11988_MOESM1_ESM.docx]

|  | **All**  **n = 221** | | **MM 0**  **n = 135** | | **MM Ia**  **n = 23** | | **MM Ib**  **n = 23** | | **MM II**  **n = 13** | | **MM III**  **n = 27** | |  |
| --- | --- | --- | --- | --- | --- | --- | --- | --- | --- | --- | --- | --- | --- |
|  | n or Median | % or IQR | n or Median | % or IQR | n or Median | % or IQR | n or Median | % or IQR | n or Median | % or IQR | n or Median | % or IQR | p |
| Age (yrs) | 66 | 56, 75 | 67 | 56, 77 | 64 | 51, 71 | 67 | 56, 76 | 60 | 55, 69 | 66 | 49, 76 | 0.359 |
| BMI (kg/m^2) | 30.1 | 27.6, 35.1 | 30 | 27.8, 5.6 | 32.2 | 28.7, 34.9 | 27.6 | 25.1, 32.5 | 34.2 | 26.2/38.4 | 31.1 | 29.3, 34.5 | 0.139 |
| Stoma Type |  |  |  |  |  |  |  |  |  |  |  |  |  |
| Ileostomy | 115 | 52.04 | 66 | 48.89 | 13 | 56.52 | 13 | 56.52 | 7 | 53.85 | 16 | 59.26 | 0.141 |
| Colostomy | 67 | 30.32 | 39 | 28.89 | 5 | 21.74 | 9 | 39.13 | 3 | 23.08 | 11 | 40.74 |  |
| Urostomy | 39 | 17.65 | 30 | 22.22 | 5 | 21.74 | 1 | 4.35 | 3 | 23.08 | 0 | 0 |  |
| Gender |  |  |  |  |  |  |  |  |  |  |  |  |  |
| Female | 116 | 52.49 | 65 | 48.15 | 14 | 60.87 | 16 | 69.57 | 8 | 61.54 | 13 | 48.15 | 0.289 |
| Male | 105 | 47.51 | 70 | 51.85 | 9 | 39.13 | 7 | 30.43 | 5 | 38.46 | 14 | 51.85 |  |
| Race |  |  |  |  |  |  |  |  |  |  |  |  |  |
| Black or African American | 5 | 2.26 | 4 | 2.96 | 1 | 4.35 | 0 | 0 | 0 | 0 | 0 | 0 | 0.891 |
| Hispanic | 2 | 0.9 | 2 | 1.48 | 0 | 0 | 0 | 0 | 0 | 0 | 0 | 0 |  |
| White | 214 | 96.83 | 129 | 95.56 | 22 | 95.65 | 23 | 100 | 13 | 100 | 27 | 100 |  |
| ASA Class |  |  |  |  |  |  |  |  |  |  |  |  |  |
| 2 | 10 | 4.52 | 4 | 2.96 | 2 | 8.7 | 2 | 8.7 | 2 | 15.38 | 0 | 0 | 0.087 |
| 3 | 201 | 90.95 | 121 | 89.63 | 21 | 91.3 | 21 | 91.3 | 11 | 84.62 | 27 | 100 |  |
| 4 | 10 | 4.52 | 10 | 7.41 | 0 | 0 | 0 | 0 | 0 | 0 | 0 | 0 |  |
| Taking Immunosuppressants |  |  |  |  |  |  |  |  |  |  |  |  |  |
| No | 196 | 88.69 | 121 | 89.63 | 22 | 95.65 | 18 | 78.26 | 13 | 100 | 22 | 81.48 | 0.148 |
| Yes | 25 | 11.31 | 14 | 10.37 | 1 | 4.35 | 5 | 21.74 | 0 | 0 | 5 | 18.52 |  |
| Current Nicotine Use |  |  |  |  |  |  |  |  |  |  |  |  |  |
| No | 201 | 91.78 | 117 | 87.97 | 23 | 100 | 21 | 91.3 | 13 | 100 | 27 | 100 | 0.084 |
| Yes | 18 | 8.22 | 16 | 12.03 | 0 | 0 | 2 | 8.7 | 0 | 0 | 0 | 0 |  |
| HTN |  |  |  |  |  |  |  |  |  |  |  |  |  |
| No | 71 | 32.13 | 44 | 32.59 | 9 | 39.13 | 6 | 26.09 | 2 | 15.38 | 10 | 37.04 | 0.577 |
| Yes | 150 | 67.87 | 91 | 67.41 | 14 | 60.87 | 17 | 73.91 | 11 | 84.62 | 17 | 62.96 |  |
| DM |  |  |  |  |  |  |  |  |  |  |  |  |  |
| No | 180 | 81.45 | 106 | 78.52 | 22 | 95.65 | 18 | 78.26 | 10 | 76.92 | 24 | 88.89 | 0.271 |
| Yes | 41 | 18.55 | 29 | 21.48 | 1 | 4.35 | 5 | 21.74 | 3 | 23.08 | 3 | 11.11 |  |
| COPD |  |  |  |  |  |  |  |  |  |  |  |  |  |
| No | 195 | 88.24 | 115 | 85.19 | 23 | 100 | 19 | 82.61 | 13 | 100 | 25 | 92.59 | 0.125 |
| Yes | 26 | 11.76 | 20 | 14.81 | 0 | 0 | 4 | 17.39 | 0 | 0 | 2 | 7.41 |  |
| Post-Op Year |  |  |  |  |  |  |  |  |  |  |  |  |  |
| 1 Year (+/- 6m) | 113 | 51.13 | 73 | 54.07 | 10 | 43.48 | 11 | 47.83 | 7 | 53.85 | 12 | 44.44 | 0.804 |
| 2 Year (+/- 6m) | 108 | 48.87 | 62 | 45.93 | 13 | 56.52 | 12 | 52.17 | 6 | 46.15 | 15 | 55.56 |  |
| Time from Operation (m) | 16 | 11, 21 | 14 | 11, 21 | 19 | 10, 22 | 19 | 11, 21 | 15 | 11, 20 | 21 | 10, 24 | 0.332 |
| Randomization |  |  |  |  |  |  |  |  |  |  |  |  |  |
| Sugarbaker | 114 | 51.58 | 67 | 49.63 | 16 | 69.57 | 12 | 52.17 | 9 | 69.23 | 10 | 37.04 | 0.131 |
| Keyhole | 107 | 48.42 | 68 | 50.37 | 7 | 30.43 | 11 | 47.83 | 4 | 30.77 | 17 | 62.96 |  |
